# A multicenter study of radiation doses to the eye lenses of clinical physicians performing radiology procedures in Japan

**DOI:** 10.1002/1348-9585.12305

**Published:** 2021-12-10

**Authors:** Keisuke Nagamoto, Takashi Moritake, Koichi Nakagami, Koichi Morota, Satoru Matsuzaki, Naoki Kunugita

**Affiliations:** ^1^ Department of Radiology Hospital of the University of Occupational and Environmental Health, Japan Kitakyushu Fukuoka Japan; ^2^ Department of Radiation Regulatory Research Group National Institute of Radiological Sciences Quantum Life and Medical Science Directorate National Institute for Quantum and Radiological Science and Technology Chiba Japan; ^3^ Department of Occupational and Community Health Nursing School of Health Sciences University of Occupational and Environmental Health, Japan Kitakyushu Fukuoka Japan; ^4^ Department of Radiology Shinkomonji Hospital Kitakyushu Fukuoka Japan

**Keywords:** eye lens dose, multiple radiation protection, occupational exposure, radiology procedures, radio‐photoluminescence glass dosimeters

## Abstract

**Purpose:**

We investigated occupational dose to the lens of the eye for physicians engaged in radiology procedures. We evaluated the potential for compliance with the new‐equivalent dose limits to the lens of the eye. Further, a “multiple radiation protection” protocol was proposed according to the basic principles of occupational health, and its effectiveness was estimated.

**Methods:**

Physicians engaged in radiology procedure at medical facilities in Japan were included in this study. The eye lens dose (3‐mm dose equivalent: H_p_(3)) for each participant was measured using a small radio‐photoluminescence glass dosimeter mounted on lead glasses. Physicians were directed to procedure multiple radiation protection measures to evaluate their usefulness.

**Results:**

The H_p_(3) was reduced by multiple radiation protection in all physicians. In particular, the H_p_(3) reduced from 207.7 to 43.2 μSv/procedure and from 21.6 to 10.2 μSv/procedure in cardiovascular internal physician and cerebrovascular physician, respectively, after the implementation of the proposed multiple radiation protection measures. The dose reduction rate of these measures was 53% (range: 37%–79%).

**Conclusions:**

The radiation doses received by the eye lenses of physicians engaged in radiology procedure may exceed the dose limits to the lens of the eye if radio‐protective equipment and imaging conditions are not properly controlled. However, based on the lens equivalent dose data, the implementation of “multiple radiation protection” according to the basic principles of occupational health can ensure compliance with the new‐equivalent dose limits to the lens of the eye without placing an undue burden on individual physicians or medical facilities.

## 1 INTRODUCTION

In April 2011, the International Commission on Radiological Protection (ICRP) Statement on Tissue Reactions (Seoul Statement), reduced the threshold dose for cataracts to 0.5 Gy and issued the following recommendation for the eye lens equivalent dose limit for occupational exposure in planned exposure situations: “for occupational exposure in planned exposure situations the Commission now recommends an equivalent dose limit for the lens of the eye of 20 mSv/year, averaged over defined periods of 5 years, with no single year exceeding 50 mSv.^1^” In response to this ICRP recommendation, the relevant Japanese national policy, Ordinance on Prevention of Ionizing Radiation Hazards, and the lens equivalent dose limit was revised from 150 to 100 mSv over 5 years and 50 mSv/year (revised in April 2021).

The occupational dose to the lens of physicians involved in radiology procedure has been reported to be significant in interventional radiology (IVR) procedures for cerebrovascular^2^, ^3^, ^4^, ^5^ and cardiovascular^3^, ^6^ medicine, tumors,^3^, ^7^ endoscopic retrograde cholangiopancreatography (ERCP),^3^, ^8^, ^9^ and orthopedic surgery.^10^ The lens equivalent dose limit is considered to have exceeded when cardiologists and gastroenterologists perform radiology procedure without radiation protection for the lens of the eye.^3^, ^6^, ^9^, ^11^ For this reason, the International Atomic Energy Agency disseminates information regarding the possibility of reducing exposure using lead glasses and ceiling‐mounted radiation shielding screens and educates the workers on the importance of eye lens protection.^12^

ICRP reported that many physicians who perform radiology procedures have inadequate radiation protection.^13^, ^14^ Although the wear rate of lead aprons and neck guards by physicians performing radiology procedures is higher than 90%,^15^, ^16^ the wear rate of lead glasses is 30%–52%.^3^, ^15^, ^16^, ^17^, ^18^ Moreover, despite the ability of lead‐containing ceiling‐mounted radiation shielding screens to reduce eye lens exposure by over 70%,^19^, ^20^, ^21^, ^22^ these screens are not always used appropriately in actual medical procedure,^17^ putting physicians at risk of receiving high radiation doses to the lens of the eye.

In this multicenter study, by applying basic principles of occupational health, we proposed “multiple radiation protection” measures that did not place an undue physical burden on physicians and were less expensive for medical facilities and estimated the effectiveness of these measures. In addition, the occupational dose to the lens of the eye for physicians was measured on a case‐by‐case basis, and the potential for compliance with the new‐equivalent dose limits to the lens of the eye (ICRP: average annual limit, 20 mSv/year over 5 years) was assessed.

## 2 METHODS

### 2.1 Participants for measurement

Between April 2019 and July 2019, 15 physicians engaged in radiology procedure (angiography, non‐angiography, or IVR procedure) at 15 medical facilities in Japan were nominated by their respective societies (Japanese Society of Radiology and the Japanese Society of Interventional Radiology, Japanese Orthopedic Association, Japanese Society of Gastroenterology, and Japanese Society of Neuroendovascular Therapy). Eye lens doses (3‐mm dose equivalent, i.e., H_p_(3)) were measured for each participant when they performed radiology procedure using conventional methods before implementing radio‐protective measures (before radiation protection measures, Table 1) and after the implementation of radio‐protective measures (after radiation protection measures, Table 1), taking into account the facility environment and the procedures in place at each medical facility. We confirmed the doctor's radiation protection method from the pre‐questionnaire and the photographs during the procedure. The personal dose values for the past 3 years and the number of procedures performed over the past year were also investigated. Since there was no evaluation of 3‐mm dose equivalent at that time, the personal dose values were defined as the 70‐µm dose equivalent of the skin or the 1‐cm dose equivalent of the effective dose, whichever is larger, as the eye lens dose.

**TABLE 1:** Radiation protection status before and after the radiation protection measures

Physician	Measures before/after	Simultaneous irradiation	Pulse rate reduction	Irradiation field	IR	Lead glasses	Shielding screens	C_u_	C_s_	R	Evacuation from the room
Cardiologist A	Before	○		○		○^a^	●^f^	○			
	After	○	○	○		○^a^	○^f^	○			
Cardiologist B	Before	○	○	○			●^g^	○			
	After	○	○	○		○^a^	○^g^	○			
Cardiologist C	Before	○	○	○		○^b^		○			
	After	○	○	○		○^c^	○^g^	○			
Neurosurgery D	Before					○^d^		○			
	After	○	○	○		○^d^	○^g^	○			
Neurosurgery E	Before			○		○^a^	●^g^	○			○
	After	○	○	○		○^a^	○^g^	○			○
Gastroenterologist F	Before			○		○^a^			●^h^		
	After		○	○		○^a^			○^h^		
Gastroenterologist G	Before										
	After					○^a^					
Gastroenterologist H	Before					○^a^			○^i^		
	After		○			○^e^			○^i^		
Gastroenterologist I	Before								●^h^		
	After					○^a^			○^h^		
Gastroenterologist J	Before								○^h^		
	After					○^a^			○^h^		
Orthopedic Surgeon K	Before				○	○^d^		●			
	After		○	○	○	○^d^		○		○	○
Orthopedic Surgeon L	Before										
	After					○^d^					
Orthopedic Surgeon M	Before										
	After					○^a^					
Radiologist N	Before		○	○	○	○^a^	○^g^	○			○
	After		○	○	○	○^a^	○^g^	○			○
Radiologist O	Before		○	○	○	○^a^	○^g^	○			○
	After		○	○	○	○^a^	○^g^	○			○

Note

○ Radioprotective equipment was being used appropriately. ● Radioprotective equipment was used, but the method of use was inappropriate. Simultaneous irradiation = reduction of simultaneous frontal and lateral irradiation in fluoroscopy mode; Pulse rate reduction = appropriate selection/switching of fluoroscopy mode (changed from 15 to 7.5 pps); Irradiation field = restriction of irradiation field to target region; IR = dose reduction using iterative reconstruction (IR) during CT fluoroscopy; Shielding screens = ceiling‐mounted radiation‐shielding screens; C_u_ = under‐bed protective curtains; C_s_ = scatter‐radiation protection curtains; R = RADPAD^®^; Evacuation from the room = room evacuation during imaging mode.

^a^ Lead glasses (Panorama shield^®^; HF‐400 ultra‐light 0.07‐mm Pb; Toray).

^b^ Lead glasses (HAGOROMO Face Guard FG06‐110; 0.06‐mm Pb; Maeda).

^c^ Lead glasses (CROSSLINK 0.75‐mm Pb; Barrier technologies^®^).

^d^ Lead glasses (Panorama shield^®^; HF‐350 ultra‐light 0.07‐mm Pb; Toray).

^e^ Lead glasses (ProTech eyewear PT‐COMET 0.75‐mm Pb; Maeda).

^f^ Ceiling‐mounted radiation shielding screen 350 (0.5‐mm Pb; Kenex).

^g^ Ceiling‐mounted radiation shielding screen (MAVIG 0.5‐mm Pb; MAVIG GmbH).

^h^ Scatter‐radiation protection curtains (Scatter protection cloth NP, 0.125‐mm Pb; Maeda).

^i^ Scatter‐radiation‐protection curtains (self‐made scatter‐protection clothing, 0.25‐mm Pb).

### 2.2 X‐ray equipment and radio‐protective methods

Of the 15 participating medical institutions, six used biplane angiography, three used hybrid single‐plane angiography combined with X‐ray computed tomography (CT), and one used surgical X‐ray fluoroscopy. The remaining five centers used X‐ray fluoroscopy systems, of which four used over‐table X‐ray tube systems and one used an under‐table X‐ray tube system.

Radiation protection measures for physicians involved in radiology procedures included the use of reduction of simultaneous front‐to‐side irradiation during fluoroscopy, appropriate selection/switching of the fluoroscopy mode (switching from 15 pps to 7.5 pps), restriction of the irradiation field to the target range, dose reduction performed using iterative reconstruction (IR) in CT fluoroscopy in combination to avoid negatively influencing radiology procedure, lead glasses, ceiling‐mounted radiation‐shielding screens, scatter‐radiation–shielding curtain for over‐table X‐ray tube systems, and under‐bed protective curtains, RADPAD^®^ (0.25 mmPb, Nippon Medical Readers Co., Ltd.), evacuation of the examination room during imaging. The lead glasses used were as follows: HF‐400 (0.07‐mm Pb‐equivalent; Toray Medical Inc., *n* = 11), HF‐350 (0.07‐mm Pb‐equivalent; Toray Medical Inc., *n* = 3), FG06‐110 (0.06‐mm Pb‐equivalent; Maeda, *n* = 1), CROSSLINK (0.75‐mm Pb; Barrier technologies^®^, *n* = 1) and PT‐COMET (0.75‐mm Pb‐equivalent; Maeda, *n* = 1). The ceiling‐mounted radiation‐shielding screens used were as follows: ceiling‐mounted radiation shield 350 (0.5‐mm Pb‐equivalent; Kenex, *n* = 1) and MAVIG (0.5‐mm Pb‐equivalent; MAVIG GmbH, *n* = 6). In addition, for the over‐table X‐ray tube systems, scatter protection was provided by NP cloth (0.125‐mm Pb‐equivalent; Maeda, *n* = 3) and facility‐made scatter‐protection curtain produced by each medical institution (0.25‐mm Pb‐equivalent, *n* = 1) (Table 1).

### 2.3 Details concerning radiation protection

We confirmed the physician's radiation protection method from the pre‐questionnaire and the photographs during the procedure. We have proposed the main protection methods based on the current situation. (Based on the three principles of external exposure protection, installing a ceiling‐mounted radiation shielding screen, reducing the pulse rate within the range that does not deteriorate the image quality, and if necessary, they were instructed to wear protective equipment.)

### 2.4 Method for measuring occupational dose to the lens of the eye

**FIGURE 1:**
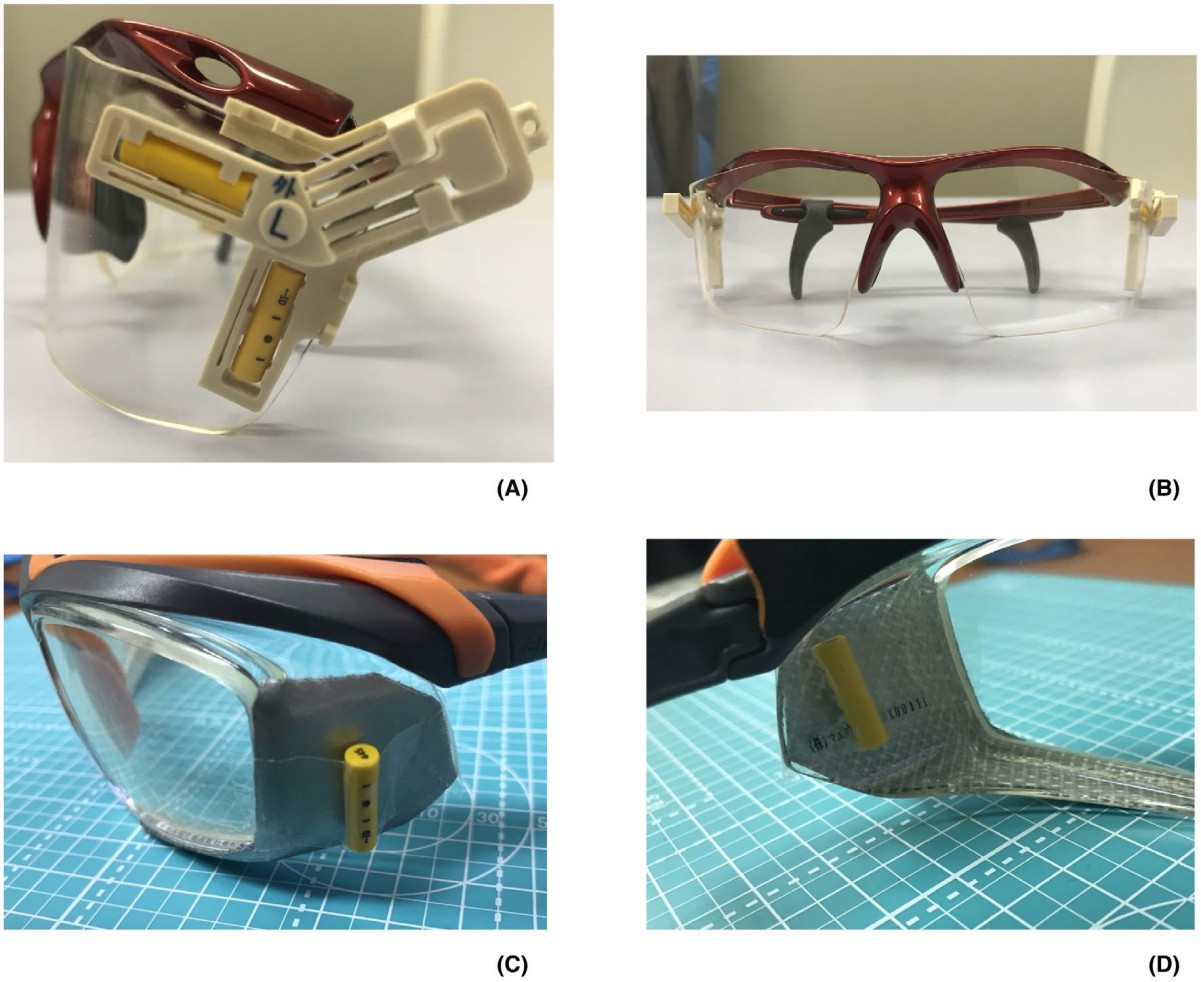
Eye lens dosimeter clip position on the lead glasses used: (A) side view, (B) front view. Location of radio‐photoluminescence glass dosimeters (RPLDs) on the lead glasses used (when tape is used): (C) side view, (D) inner side view. Four RPLDs are placed on the left and right sides of the lead glasses

Physicians wore lead aprons and lead glasses. The H_p_(3) to the lens of the eye was obtained from air kerma measurements obtained by radio‐photoluminescence glass dosimeters^23^ (GD‐352M; Chiyoda Technol) attached to the inner and exterior sides of the lead glasses. The GD‐352M used in the measurements complied with the IEC62387 requirements for dosimetry systems with passive detectors and provided stable dose linearity in the low dose range (less than ±5.0% in the range of 0.01 to 50 mGy).^23^, ^24^ Before the start of this study, the coefficient of variation was confirmed to not exceed 3.0%. We modified a previously reported eye lens dosimeter clip^25^ component and attached it to the left and right sides of the lead glasses and placed one GD‐352M unit each in fixed positions on the inner and outer sides of the lens (Figure 1A,B). For lead glasses where the eye lens dosimeter clips could not be used, GD‐352M units were attached to the left and right sides of the lens using adhesive tape (Figure 1C,D).

After the measurements were completed, the radio‐photoluminescence glass dosimeters were stored in a low‐background area outside the radiation‐controlled area and returned to the providing university by postal mail after the survey period. The data were then read and analyzed using a reading device (FGD‐1000; Chiyoda Technol) installed at our institution.

The eye lens dose H_p_(3) in this study was calculated from air kerma measurements obtained using the radio‐photoluminescence glass dosimeters. Specifically, the air kerma to H_p_(3) conversion coefficient K (H_p_(3)/air kerma) on a cylindrical phantom (φ20 cm × 20 cm) was calculated using a Monte Carlo simulation from a previous report,^26^ and the conversion was performed according to Equation (1). In this study, the effective energy used in radiology procedure was assumed to be 50 keV, and 1.590 Sv/Gy was adopted for K (H_p_(3)/air kerma):

(1) $$H_{p}\left(3\right)=K\cdot \text{air}\;\text{kerma},$$

where H_p_(3) is the lens dose of the physicians eye per procedure (μSv), air kerma is the radio‐photoluminescent glass dosimeter measured value (μGy), *K* is the air kerma to H_p_(3) conversion coefficient (H_p_(3)/air kerma) (Sv/Gy).

For each physician, the H_p_(3) values measured before the radiation protection measures were compared between the left and right eyes, and the value indicating a greater dose was recorded as the H_p_(3) in this study. For evaluations after the radiation protection measures, the H_p_(3) values for the dose on the same side as that measured before the measures were recorded.

### 2.5 Method for calculating H_p_(3)_rate_ to the lens of the eye

To determine the lens dose of the eye per unit time, the H_p_(3) values obtained in Equation (1) were divided by the fluoroscopy time of the procedure to obtain the eye lens dose rate per unit time (H_p_(3)_rate_) according to Equation (2) below:

(2) $$H_{p}{\left(3\right)}_{\text{rate}}=H_{p}\left(3\right)/T,$$

where H_p_(3)_rate_ is the lens dose of the eye per unit time (μSv/min), H_p_(3) is the lens dose of the physicians’ eye per procedure (μSv), *T* is the fluoroscopy time during the procedure (min).

### 2.6 Calculation of number of possible annual radiology procedures by physicians

The median value of lens dose of the physicians’ eye (H_p_(3)_procedure_) was calculated from the median value of the H_p_(3)_rate_ calculated in Equation (2) and the median fluoroscopy time of the procedures performed before and after the radiation protection measures. The number of possible annual radiology procedures was obtained based on Equations (3) and (4), respectively:

(3) $$H_{p}{\left(3\right)}_{\text{procedure}}=H_{p}{\left(3\right)}_{\text{rate}\;\text{median}}\cdot T_{\text{median}},$$

(4) $$\text{Number}\;\text{of}\;\text{possible}\;\text{annual}\;\text{radiology}\;\text{procedures}=H_{\text{lens}}/H_{p}{\left(3\right)}_{\text{procedure}},$$

where H_p_(3)_procedure_ is the median value of lens dose of the physicians eye (μSv/procedure), H_p_(3)_rate median_ is the median value of eye lens dose rate per unit time (μSv/min), *T*_median_ is the median value of fluoroscopy time before or after radiation protection measures (min), H_lens_ is the new‐equivalent dose limits to the lens of the eye (ICRP: average annual limit, 20 mSv/year over 5 years).

### 2.7 Analysis of the physician lens dose reduction rate with before and after radiation protection measures

To determine the dose reduction effect of radiation protection measures, the dose reduction rate (DRR) attributable to measures was calculated from before the measures H_p_(3)_procedure_ (H_p_(3)_procedure before_) and the H_p_(3) after the measures (H_p_(3)_procedure after_) according to Equation (5).

(5) $$\text{DRR}=(1‐H_{p}{\left(3\right)}_{\text{procedure}\;\text{after}}/H_{p}{\left(3\right)}_{\text{procedure}\;\text{before}})\times 100,$$

where DRR is the dose‐reduction rate, H_p_(3)_procedure before_ is before the measures H_p_(3)_procedure_ (μSv/procedure), H_p_(3)_procedure after_ is after the measures H_p_(3)_procedure_ (μSv/procedure).

### 2.8 Statistical analysis

Differences in H_p_(3)_rate_ before and after the radiation protection measures were confirmed using the Kruskal–Wallis one‐way analysis of variance. When the one‐way analysis of variance result was significant, the difference between the individual ‐before‐ and ‐after‐ radiation protection measures was evaluated using the Dunn test (with Bonferroni correction). Differences in fluoroscopy time before and after the radiation protection measures were confirmed using the Mann–Whitney *U* test. Differences were considered statistically significant at *P* < .05. All analyses were performed using the Statistical Package for the Social Science (version 25.0, IBM Corporation).

### 2.9 Ethical considerations

The study was approved by the Ethics Committee of the University of Occupational and Environmental Health, Kitakyushu, Japan (Protocol Number R1‐054).

## 3 RESULTS

One orthopedic surgeon who had incorrectly installed an eye lens dosimeter, lost a radio‐photoluminescence glass dosimeter, and failed to keep a dosimeter was excluded from the analysis, and the data from the remaining 14 physicians were analyzed.

### 3.1 Eye lens dose over the previous 3 years (past personal dose information)

Past personal dose information concerning the physicians eye lens dose is shown in Table 2. Since there was no evaluation of 3‐mm dose equivalent at that time, the personal dose values were defined as the 70‐µm dose equivalent of the skin or the 1‐cm dose equivalent of the effective dose, whichever is larger, as the eye lens dose. The proportion of doses exceeding the new‐equivalent dose limits to the lens of the eye was 27% (4/14). Particularly high H_p_ values were reported for cardiologist A and gastroenterologist F (42.3 and 75.3 mSv/year, respectively).

**TABLE 2:** Occupational dose to the lens of the eye for over the past 3 years by physicians

Physicians	FY2016	FY2017	FY2018
Cardiologist A	**49.8**	**51.7**	**42.3**
Cardiologist B	18	15.2	**31.0**
Cardiologist C	N/A^a^	N/A^a^	N/A^a^
Neurosurgeon D	14.5	12.8	11.9
Neurosurgeon E	12.1	14.5	12.7
Gastroenterologist F	**22.1**	**21.4**	**75.3**
Gastroenterologist G	N/A^a^	N/A^a^	3.6
Gastroenterologist H	0.9	0.0	0.3
Gastroenterologist I	8.9	15.3	9.9
Gastroenterologist J	N/A^c^	N/A^c^	**27.1**
Orthopedic Surgeon K	N/A^c^	0.5	0.8
Orthopedic Surgeon L	N/A^a^	N/A^a^	N/A^a^
Orthopedic Surgeon M	N/A^b^	N/A^b^	N/A^b^
Radiologist N	N/A^c^	6.0	3.9
Radiologist O	N/A^c^	N/A^c^	9.0

Note

Equivalent dose limits for the lens of the eye >20 mSv/year are in boldface. H_p_(0.07) = 70‐μm dose equivalent; FY = *fiscal year*.

^a^ Personal dosimeters had not been distributed by the hospital.

^b^ Personal dosimeters had been distributed by the hospital but were not being used.

^c^ No information on radiation dose at other hospitals before joining the hospital staff.

### 3.2 Number of radiology procedures and fluoroscopy time during which eye lens dosimetry was performed

The numbers of radiology procedures in which eye lens dosimetry measurements were taken were 5 ± 2 before the measures and 5 ± 2 after the measures (Table S1). Moreover, the fluoroscopy times recorded during the monitoring period were 10.3 (range: 0.4–114.8) min before the measures and 12.4 (range: 0.3–80.8) min after the measures. We performed the parametric test with fluoroscopy time before and after the measures and the result did not show any significant difference (*P* = .466, Mann–Whitney *U* test).

### 3.3 Details concerning radiation protection

Table 1 shows the status of radiation protection in each physicians before and after the radiation protection measures. We confirmed the physicians radiation protection method from the pre‐questionnaire and the photographs during the procedure. In assessments of the usage rate of lead glasses, 64% (9/14) of the physicians used lead glasses before the measures and 100% (14/14) did so after the measures. In this study, seven of the 14 facilities had a ceiling‐mounted radiation shielding screen. Although 29% (2/7) of the participants properly used a ceiling‐mounted radiation shielding screen before the measures, 100% (7/7) did so after the measures. With respect to the fluoroscopy devices used by gastroenterologists, 80% (4/5) used over‐table X‐ray tubes, whereas a scatter‐protection curtain was used by 100% (4/4) of the physicians to shield scatter radiation from patients.

### 3.4 Eye lens dose of physicians involved in radiology procedures

Median the H_p_(3)_rate_ of all physicians who participated in the study was reduced by the radiation protection measures. In particular, median the H_p_(3)_rate_ significantly reduced after the measures in the field of cardiovascular internal medicine (*P* = .011, Kruskal–Wallis test, Table 3) and neurology (*P* < .01, Kruskal–Wallis, Table 3). In contrast, the fluoroscopy time showed no differences among radiology procedure.

**TABLE 3:** Lens dose rate H_p_(3) and eye lens fluoroscopy time before and after radiation protection measures

Physician	Before (After)^a^	L_g_ Y/N	H_p_(3)_rate_ [μSv/min]			ANOVA^b^ (*P* value)	Fluoroscopy time [min]		*P* value ^c^
			Before‐measures		After‐measures		Before‐measures	After‐measures	
			Outside	Inside	Inside				
			Median [range]	Median [range]	Median [range]		Median [range]	Median [range]	
Cardiologist A	9 (9)	Y	12.0 [3.1–72.8]	5.2 [1.4–32.5]	2.6 [0.6–11.5]		15.3 [4.5–25.4]	17.7 [8.6–34.4]	
Cardiologist B	5 (2)	N	7.6 [2.0 –23.1]	3.9 [0.7–23.7]	2.8 [0.7–4.9]		23.2 [13.6–36.6]	29.4 [22.1–36.6]	
Cardiologist C	2 (5)	Y	23.5 [13.7–33.3]	13.4 [6.4–20.4]	1.5 [0.8–37.9]		15.5 [12.5–18.5]	28.8 [7.3–44.1]	
Circulatory Internal Medicine	16 (16)		**12.0** * **[2.0–72.8]^†^**	**5.0** * **[0.7–32.5]**	**2.6** * **[0.6–38.0]^†^**	.**011**	17.2 [4.5–36.6]	23.2 [7.3–44.1]	.363
Neurosurgeon D	5 (5)	Y	7.8 [1.3–16.7]	3.6 [1.4–7.1]	0.8 [0.5–2.2]		35.0 [20.3–84.4]	39.8 [26.9–79.3]	
Neurosurgeon E	5 (5)	Y	5.0 [4.0–11.6]	2.1 [1.5–5.3]	0.5 [0.4–1.0]		10.3 [9.2–32.9]	20.4 [10.9–21.9]	
Cerebrovascular Medicine	10 (10)		**5.0** * **[1.3–16.7]^‡^**	**2.5** * **[1.4–7.1]^§^**	**0.7** * **[0.4–2.2]^‡§^**	**<.01**	25.9 [9.2–84.4]	24.4 [10.9–79.3]	.705
Gastroenterologist F	5 (5)	Y	6.6 [4.2–14.6]	3.7 [2.2–7.7]	1.4 [0.6–4.9]		13.8 [10.1–63.9]	15.1 [6.9–28.8]	
Gastroenterologist G	4 (4)	N	1.1 [0.6–3.2]	—	0.6 [0.33–1.5]		22.7 [9.5–56.0]	22.7 [9.5–56.0]	
Gastroenterologist H	3 (3)	Y	0.4 [0.0–0.9]	0.2 [0.2–0.4]	0.0 [0.0–1.3]		2.5 [1.4–2.6]	2.0 [1.8–2.5]	
Gastroenterologist I	5 (5)	N	8.3 [3.65–12.3]	—	5.1 [2.0–9.1]		3.0 [2.0–33.0]	3.0 [2.0–33.0]	
Gastroenterologist J	5 (5)	N	2.2[0.6–3.2]	—	1.2 [0.1–2.3]		5.3 [1.9–10.8]	5.3 [1.9–10.8]	
Gastroenterological Medicine	22 (22)		2.7 [0.0–15.0]	2.5 [0.2–8.0]	1.3 [0.0–4.9]	.073	9.3 [1.4–63.9]	8.8 [1.8–56.0]	.743
Orthopedic Surgeon K	10 (7)	Y	9.0 [0.0–41.9]	8.0 [0.0–19.8]	3.1 [0.0–13.7]		1.1 [0.4–3.2]	0.6 [0.3–3.1]	
Orthopedic Surgeon L	5 (5)	N	2.9 [0.6–4.9]	—	1.1 [0.6–1.7]		6.6 [2.4–7.3]	6.6 [2.4–7.3]	
Orthopedic Surgery	15 (12)		6.4 [0.0–41.9]	8.0 [0.0–19.8]	2.5 [0.0–13.7]	.194	2.0 [0.4–7.3]	1.6 [0.3–7.3]	.807
Radiologist N	6 (6)	Y	1.9 [0.4–4.0]	1.8 [0.5–1.4]	0.7 [0.0–4.0]		48.8 [8.2–114.8]	40.6 [10.5–80.8]	
Radiologist O	6 (4)	Y	1.4 [0.2–5.6]	0.7 [0.0–2.8]	0.3 [0.0–1.0]		12.6 [1.8–30.8]	17.3 [0.3–43.0]	
Radiological Medicine	12 (10)		1.9 [0.2–5.6]	0.8 [0.0–2.8]	0.4 [0.0–4.0]	.219	21.5 [1.8–114.8]	36.4 [0.3–80.8]	.997

Note

The significant difference (*P* < .05.) is set in boldface.

L_g_ = lead glasses; Y/N = wear glasses/do not wear glasses; H_p_(3)_rate_ = lens dose of the eye per unit time (3‐mm dose equivalent).

^a^ Number of Before‐measures (After‐measures) procedures.

^b^ Differences in H_p_(3)_rate_ before and after the radiation protection measures were confirmed using the Kruskal–Wallis one‐way analysis of variance. When the one‐way analysis of variance result was significant, the difference between the individual before and after radiation protection measures was evaluated using the Dunn test (with Bonferroni correction).

^c^ Differences in fluoroscopy time before and after the radiation protection measures were confirmed using the Mann–Whitney *U* test. The same symbols indicate that Bonferroni‐corrected Dunn test multiple comparisons showed significant differences. All analyses were performed using the Statistical Package for the Social Science (version 25.0, IBM Corporation).

* *P* < .05.

### 3.5 Annual number of cases indicated for radiology procedure per physician

Based on the new‐equivalent dose limits to the lens of the eye and the H_p_(3)_procedure before_ (μSv/procedure), the number of cases indicated for radiology procedure per year was calculated, and this number was lower than the number of radiology procedures performed before the radiation protection measures in fiscal year 2018 (FY2018) for three physicians (Table 4). However, after the implementation of the proposed radiation protection method (Table 1), the number of cases indicated for radiology procedure exceeded the number of radiology procedures performed in FY2018 for all physicians (Table 4). The DRR of this study was 53% (range: 37%–79%).

**TABLE 4:** Eye lens dose reduction rate in radiation protection measures and number of possible annual radiology practices by clinical department

Physician	Number of radiology practice	H_p_(3)_procedure_ (μSv/procedure)		DRR^c^ [%]	Number of possible annual radiology practices	
	FY2018	H_p_(3)_procedure before_ ^a^	H_p_(3)_procedure after_ ^b^		Before‐measures^d^	After‐measures^e^
Cardiologist A	421	79.6	46.0	42	**251**	435
Cardiologist B	26	176.3	82.3	53	113	243
Cardiologist C	154	207.7	43.2	79	**96**	463
Neurosurgeon D	120	126.0	79.0	37	159	253
Neurosurgeon E	120	21.6	10.2	53	925	1961
Gastroenterologist F	397	51.1	21.1	59	**392**	946
Gastroenterologist G	40	24.8	13.5	45	807	1479
Gastroenterologist H	70	1.0	N/A ^f^	—	20 000	—
Gastroenterologist I	111	24.9	15.3	39	803	1307
Gastroenterologist J	304	11.7	6.4	46	1705	3145
Orthopedic Surgeon K	97	8.8	2.0	78	2273	10 187
Orthopedic Surgeon L	Unknown	19.1	7.3	62	1045	2755
Radiologist N	202	87.8	28.4	68	228	704
Radiologist O	222	8.8	5.2	41	2268	3854

Note

Number of radiology practice FY2018 > number of possible radiology practices per year are set in boldface; FY2018 = fiscal year 2018; DRR = dose reduction rate.

^a^ The median value of eye lens dose before radiation protection measures.

^b^ The median value of eye lens dose after radiation protection measures.

^c^ The dose‐reduction rate (DRR) [%] = (1 − *b*/*a*) 100.

^d^ Number of possible annual radiology practices (before radiation protection measures) = The equivalent dose limits to the lens of the eye (ICRP: average annual limit, 20 mSv/year over 5 years) /*a*.

^e^ Number of possible annual radiology practices (after radiation protection measures) = The equivalent dose limits to the lens of the eye (ICRP: average annual limit, 20 mSv/year over 5 years)/*b*.

^f^ Less than the lower limit of dose measurement.

### 3.6 Case 1: Radiation protection measures for neurosurgeons

**FIGURE 2:**
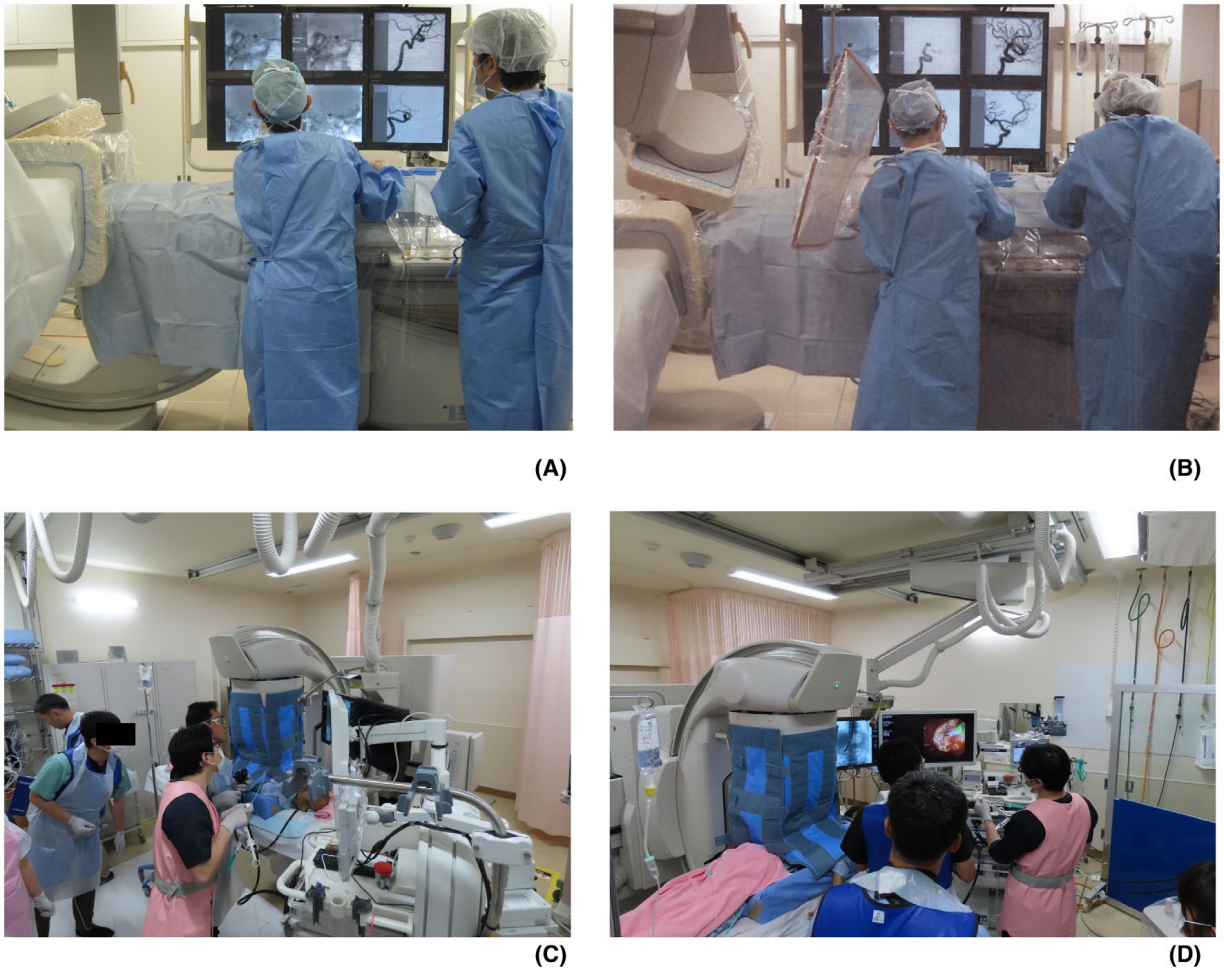
Photographs of a physician in the field of cardiovascular internal medicine performing an interventional radiology procedure. (A) Radiation protection before the radiation protection measures: No ceiling‐mounted radiation shielding screen. (B) Radiation protection after radiation protection measures: A ceiling‐mounted radiation shielding screen has been used. (C) Photographs of a physician in the field of gastroenterological internal medicine performing an endoscopic retrograde cholangiopancreatography. A scatter‐protection cloth developed for the over‐table X‐ray tube has been used. (D) Photographs of a physician in the field of gastroenterological internal medicine performing an endoscopic retrograde cholangiopancreatography. A scatter‐protection cloth developed for the over‐table X‐ray tube has been used

The number of IVRs in the field of head and neck medicine performed by neurosurgeon D, was 120 in FY2018 (Table 4). The equivalent eye lens dose calculated from the personal dosimeter attached to the neck was 11.9 mSv/year (Table 2), which was within the new‐equivalent dose limits to the lens of the eye. Even if neurosurgeon D maintains the dose (11.9 mSv/year), the total working period of 50 years is 595 (11.9 mSv/year × 50 years) mSv, which exceeds the radiation cataract threshold of 500 mGy. Therefore, it is necessary to adhere to the dose limit and optimize it in addition. As such, the radiation protection status of neurosurgeon D was investigated (Table 1). We found that although lead glasses were used, a ceiling‐mounted radiation shielding screen was not (Figure 2A). In addition, this physician performed procedures with simultaneous frontal and lateral irradiation and a high pulse rate (15 pps) during fluoroscopy. As such, the H_p_(3)_rate median_ before the measures was 3.6 μSv/min. The H_p_(3)_procedure before_ was 126.0 μSv/procedure based on this H_p_(3)_rate median_, and the median fluoroscopy time was 35.0 min (median) before radiation protection measures. The resultant 159 IVR procedures targeting the head and neck region would exceed the new‐equivalent dose limits to the lens of the eye.

As such, we urged neurosurgeon D to use a ceiling‐mounted radiation shielding screen and to reduce the radiation dose (i.e., to reduce the simultaneous irradiation of the front side during fluoroscopy and to reduce the pulse rate during fluoroscopy to 7.5 pps), and the eye lens dose H_p_(3) on the inner side of the lead glasses was evaluated under these conditions (Figure 2B). As a result, the H_p_(3)_rate median_ after the radiation protection measures was 0.8 μSv/min, and the H_p_(3)_procedure before_ was 79.0 μSv/procedure, calculated from the fluoroscopy time of 15.1 min (median) after the radiation protection measures. This indicates that 253 IVR procedures at the head and neck region could be performed, and the possibility of exceeding the new‐equivalent dose limits to the lens of the eye was low, even considering the 120 procedures performed in FY2018.

### 3.7 Case 2: Radiation protection measures for gastroenterologists

Gastroenterologist F, performed 397 radiology examinations as a part of ERCP procedures in FY2018 (Table 4). The eye lens equivalent dose calculated from the personal dosimeter attached to the neck was 75.3 mSv/year (Table 2), indicating that the exposure with the current radiation protection method exceeded the new‐equivalent dose limits to the lens of the eye. We investigated the radiation protection status of Gastroenterologist F (Table 1) and found that although the physician used scatter‐protection curtain and lead glasses, there may have been inadequacies in the use of scatter‐protection curtain. Moreover, gastroenterologist F was found to be performing the procedures at a high pulse rate (15 pps). As such, we measured the H_p_(3)_rate median_ before the radiation protection measures and found that it was 3.7 μSv/min., which was the highest among participating gastroenterologists using over‐table X‐ray tubes. Since the H_p_(3)_procedure before_ obtained from this H_p_(3)_rate median_ value and the median fluoroscopy time of 13.8 min before the radiation protection measures was 51.1 μSv/procedure, the new equivalent dose limits to the lens of the eye could be exceeded after 392 ERCP procedures. Therefore, we evaluated the H_p_(3) on the inner side of the lead glasses after explaining the proper use of scatter‐protection curtain (Figures 2C,D) to gastroenterologist F and urging this physician to reduce the radiation dose by reducing the pulse rate during fluoroscopy to 7.5 pps. Subsequently, the H_p_(3)_rate median_ inside the lead glasses was 1.4 μSv/min, and the H_p_(3)_procedure before_ was 21.1 μSv/procedure, calculated from the median fluoroscopy time of 15.1 min after the radiation protection measures. At this exposure, 946 ERCP procedures could be performed under the new‐equivalent dose limits to the lens of the eye, and the possibility of exceeding the new equivalent dose limits to the lens of the eye was low even when considering the 397 procedures performed in FY2018.

## 4 DISCUSSION

In this multicenter study, we investigated the H_p_(3) of physicians on a case‐by‐case basis and evaluated the potential for compliance with the new‐equivalent dose limits to the lens of the eye. In addition, we proposed “multiple radiation protection” measures for physicians engaged in radiology procedure according to the basic principles of occupational health and showed that adherence to these measures would ease compliance with the new‐equivalent dose limits to the lens of the eye without imposing an excessive burden on physicians or medical facilities.

Many physicians in the field of radiology perform procedures without adequate training and knowledge of radiation protection.^13^, ^14^ In this study, radiation protection was properly implemented only during procedures performed by radiologists who specialized in diagnostic imaging using radiation, and procedures performed in non‐radiology departments showed inadequate implementation of radiation protection measures (Table 1). The usage rate of lead glasses increased from 64% (9/14) to 100% (14/14) after‐measures. This result is higher than that reported in previous studies (30%–52%).^3^, ^15^, ^17^, ^18^ In addition, 50% (7/14) of the facilities had ceiling‐mounted radiation shielding screens, but only 29% (2/7) of the physicians (all physicians were radiologists) were able to use these screens appropriately. Although the shielding effect of the lead‐containing ceiling‐mounted radiation shielding screens is known to be high,^19^, ^20^, ^21^, ^22^ cardiologists and neurosurgeons in our study were unable to use the radiation shielding screens properly—the same as previous reports.^17^ Therefore, we created a video explaining the proper use of ceiling‐mounted radiation shielding screens and encouraged the use of this protective equipment in close contact with patients (Video S1). After the measures, 100% (7/7) of the physicians used the ceiling‐mounted radiation shielding screen in an appropriate configuration. In addition, despite the fact that the scatter‐protection curtain used in gastroenterology procedure has been reported to have a high shielding effect,^27^, ^28^ some gastroenterologists who used scatter‐protection curtain had a high H_p_(3)_rate median_. As a factor, the shielding effect may be reduced if the scatter‐protection curtain is pulled up during fluoroscopy to observe a patient's chest movement.^27^, ^28^ Since monitoring of the patient's respiratory status is essential in procedures such as ERCP, during which the patients are sedated, we recommend the use of devices such as pulse oximeters for respiratory management in such cases.

The H_p_(3)_rate median_ decreased in all medical fields. In particular, in the cardiovascular internal medicine (*P* = .011, Kruskal–Wallis, Table 3) and head and neck medicine (*P* < .01, Kruskal–Wallis, Table 3) fields, the H_p_(3)_rate median_ decreased significantly after the implementation of our proposed radiation protection measures. In addition to the appropriate use of a ceiling‐mounted radiation shielding screen (Figure 2B), the following measures were employed to reduce the overall radiation dose and significantly reduce the H_p_(3): avoiding simultaneous front‐to‐side irradiation during fluoroscopy, using a low pulse rate (7.5 pps) to the extent that it did not affect radiology procedures, and restricting the irradiation field to the target imaging range. The H_p_(3)_rate median_ before and after the radiation protection measures in the field of orthopedic surgery were 6.4 and 2.5 μSv/min, respectively (Table 3), indicating a certain dose rate reduction effect (*P* = .194, Kruskal–Wallis, Table 3). The H_p_(3)_rate median_ in the field of orthopedic surgery was higher than that for gastroenterologists, who are known to have higher occupational eye lens doses, and the dose rate was comparable to that of cardiologists.

Radiation protection for physicians engaged in radiology procedures should be considered according to the three basic management principles of occupational health.^29^ In this study, from the viewpoint of working environment management, we proposed the following measures: reducing the use of simultaneous front‐to‐side irradiation during fluoroscopy, appropriate selection/switching of the fluoroscopy mode (from 15 to 7.5 pps), restricting the irradiation field to the target area, and dose reduction using IR in CT fluoroscopy. Next, from the viewpoint of working management, we proposed the use of radiation protection equipment such as lead glasses, ceiling‐mounted radiation shielding screens, under‐bed protective curtains, and scatter radiation protection curtain and evacuation of the room during imaging. The implementation of the “multiple radiation protection” measures, which represented a combination of working environment management and working management protocols, did the DRR by 53% (range: 37%–79%) without placing any extraordinary burden on either medical staff or medical facilities. The number of cases indicated for radiology procedure calculated from the H_p_(3)_procedure after_ [μSv/procedure] after the radiation protection measures also suggested that all physicians could comply with the new eye lens dose equivalent (Table 4). Incorporating the three areas of the fundamentals of industrial health management into the concept of radiation protection measures can effectively reduce eye lens dose without overburdening medical facilities or individuals. These results clearly suggest that physicians eye lens doses can be significantly reduced by providing appropriate advice on radiation protection. However, to implement “multiple radiation protection,” radiologists, who are actually responsible for radiation exposure control in the medical field, must actively intervene to ensure compliance with radiation protection protocols and to improve the occupational health environment.

## 5 CONCLUSION

A case‐by‐case evaluation of the eye lens dose H_p_(3) of physicians involved in radiology procedure revealed that 21% (3/14) of the physicians would be exposed to eye lens doses higher than the new‐equivalent dose limits to the lens of the eye if they performed the same number of radiology procedures as they did in FY2018 during their before‐measures radiation protection status. However, the findings also indicated that by adhering to the basic principles of occupational health, implementing the principle of “multiple radiation protection” through the review of fluoroscopy procedures and pulse rates, and using ceiling‐mounted radiation shielding screens, scatter‐protection curtains, and lead glasses, compliance with the new‐equivalent dose limits to the lens of the eye (ICRP: average annual limit, 20 mSv/year over 5 years) could be achieved without imposing an undue burden on either the physician or the medical facility.

## DISCLOSURE

*Approval of the research protocol:* The study was approved by the Ethics Committee of the University of Occupational and Environmental Health, Kitakyushu, Japan (Protocol Number R1‐054). *Informed consent:* N/A. *Registry and the registration no. of the study/trial:* N/A. *Animal studies:* N/A. *Conflict of interest:* The authors declare that there is no conflict of interest.

## AUTHOR CONTRIBUTIONS

**Keisuke Nagamoto:** Conceptualization, methodology, data curation, writing ‐ original draft, writing ‐ review & editing. **Takashi Moritake:** Conceptualization, formal analysis, writing ‐ review & editing, project administration, funding acquisition. **Koichi Nakagami:** Validation, investigation, visualization. **Koichi Morota:** Validation, data curation. **Satoru Matsuzaki:** Validation, investigation. **Naoki Kunugita:** Supervision, funding acquisition.

## Supporting information

Tabe S1

Video S1

## Data Availability

On reasonable request, derived data supporting the findings of this study are available from the corresponding author.
