# Supplementary material for: A multicenter study of radiation doses to the eye lenses of clinical physicians performing radiology procedures in Japan
Source: J Occup Health. 2021 Dec 10;63(1):e12305. doi: 10.1002/1348-9585.12305 (PMC8662660; doi:10.1002/1348-9585.12305)
Supplement: Supplementary file 1 — Tabe S1 [file JOH2-63-e12305-s001.docx]

| Supplement Table 1 Radiology procedures performed during the study period | | |
| --- | --- | --- |
|  |  | |
| Field of treatment | | Procedure |
| Circulatory Internal Medicine | | Coronary angiography (CAG) |
|  |  | Percutaneous coronary intervention (PCI) |
|  |  | Percutaneous renal artery angioplasty (PTRA) |
|  |  | Peripheral vascular intervention (endovascular treatment [EVT]) |
| Cerebrovascular Medicine | | Percutaneous transluminal angioplasty (PTA) |
|  |  | Cerebral angioembolization (AN) |
|  |  | Cerebrovascular coil embolization |
|  |  | Cerebrovascular diagnostic angiography |
|  |  | Cerebrovascular thrombus retrieval |
|  |  | Head and neck stenting (carotid artery stenting [CAS]) |
| Gastroenterological Internal Medicine | | Endoscopic retrograde cholangiopancreatography (ERCP) |
|  |  | Endoscopic papillary sphincterotomy (EST) |
|  |  | Endoscopic papillary balloon dilation (EPBD) |
|  |  | Endoscopic injection sclerotherapy (EIS) |
|  |  | Transnasal pancreatic duct drainage |
|  |  | Bile duct stenting |
| Orthopedic Surgery | | Intervertebral disc nucleus pulposis |
|  |  | Nerve root block |
|  |  | Myelography |
|  |  | Microendoscopic discectomy (MED) |
|  |  | Posterior lumbar interbody fusion (PLIF) |
|  |  | Infected pseudoarthroplasty surgery |
|  |  | Open reduction and internal fixation of fractures |
|  |  | Open reduction and internal fixation of fractures (external fixation) |
| Radiology Medicine | | CV port implantation |
|  |  | Partial hepatic artery embolization |
|  |  | Microwave ablation with hepatic dynamic chemotherapy |
|  |  | Hepatic artery chemoembolization |
|  |  | Transarterial chemoembolization (TACE) |
|  |  | Bland-TAE (Embosphere) |
